# Electrochemical Production of Glycolic Acid from Oxalic Acid Using a Polymer Electrolyte Alcohol Electrosynthesis Cell Containing a Porous TiO_2_ Catalyst

**DOI:** 10.1038/s41598-017-17036-3

**Published:** 2017-12-12

**Authors:** Masaaki Sadakiyo, Shinichi Hata, Xuedong Cui, Miho Yamauchi

**Affiliations:** 1 International Institute for Carbon-Neutral Energy Research (WPI-I2CNER), Kyushu University, 744 Moto-oka, Nishi-ku Fukuoka, 819-0395 Japan; 2 Department of Chemistry, Faculty of Science, Kyushu University, 744 Moto-oka, Nishi-ku Fukuoka, 819-0395 Japan

## Abstract

A liquid flow-type electrolyser that continuously produces an alcohol from a carboxylic acid was constructed by employing a polymer electrolyte, named a polymer electrolyte alcohol electrosynthesis cell (PEAEC). Glycolic acid (GC, an alcoholic compound) is generated on anatase TiO_2_ catalysts via four-electron reduction of oxalic acid (OX, a divalent carboxylic acid), accompanied with water oxidation, which achieves continuous electric power storage in easily stored GC. Porous anatase TiO_2_ directly grown on Ti mesh (TiO_2_/Ti-M) or Ti felt (TiO_2_/Ti-F) was newly fabricated as a cathode having favourable substrate diffusivity. A membrane-electrode assembly composed of the TiO_2_/Ti-M, Nafion 117, and an IrO_2_ supported on a gas-diffusion carbon electrode (IrO_2_/C) was applied to the PEAEC. We achieved a maximum energy conversion efficiency of 49.6% and a continuous 99.8% conversion of 1 M OX, which is an almost saturated aqueous solution at room temperature.

## Introduction

Development of electrolysers converting electricity into chemical energy is one of the current topics in energy-related chemistry^1–4^. An important role of the electrolyser is the smoothing of intermittent electric power generated using renewable energies through a temporary storage of the electricity in chemicals. So far, hydrogen gas, produced by water electrolysers^5^, has been considered to be a ubiquitous chemical for energy storage, namely an energy carrier^6,7^. However, gaseous and chemically active hydrogen involves various difficulties in storage and distribution because of its low volumetric energy density (liquefied only below 33 K)^8^, explodability^9^, and high permeability for metals^10^. On the other hand, some liquid chemicals such as methylcyclohexane^11^, formic acid^12^, and hydrogen peroxide^13^ are attracting much attention as energy carriers having considerably higher energy density under ambient conditions. Alcohols have recently emerged as a candidate of an energy carrier storing natural energies because of their low explodability and low corrosiveness, in addition to the high energy density in a liquid or solution state^14^. However, there is a lack of reports on electrocatalysts to synthesize the alcohols especially from their oxidized species, such as aldehydes and carboxylic acids. On another front, alcohol from CO_2_ gas has been widely investigated in recent years^15^. The wide distribution of products is a drawback of the CO_2_ reduction.

**Figure 1:**
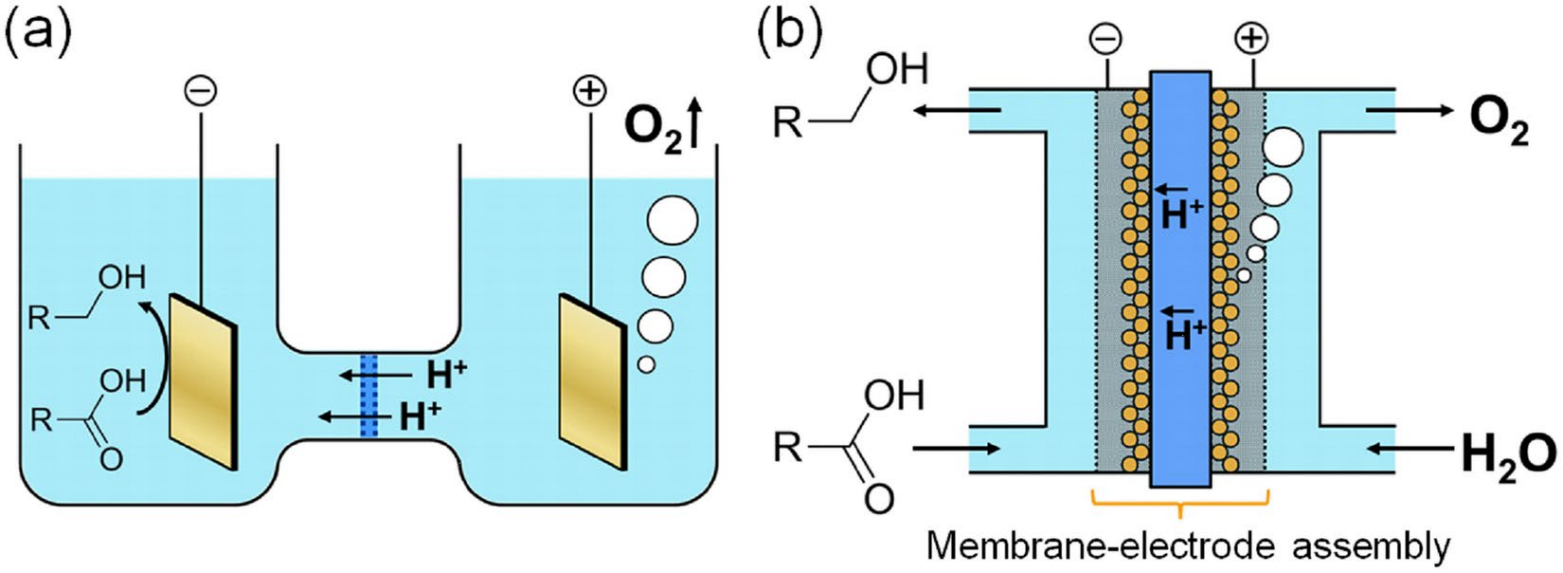
Schematic views of two types of electrolyzers for alcohol electrosynthesis from carboxylic acids. (**a**) Two-compartment alcohol electrosynthesis cell. (**b**) PEAEC for alcohol electrosynthesis from carboxylic acid.

We have especially focused on efficient electrosynthesis of alcohols from ubiquitous oxidized chemicals, i.e., carboxylic acids, for efficient power storage. Recently, we succeeded in electroreduction of oxalic acid (OX, a divalent carboxylic acid) to produce glycolic acid (GC, a monovalent alcohol) through four-electron reduction (Fig. S1, in the Supplementary Information) of the acid on the cathode placed in a two-compartment electrochemical cell (Fig. 1a) with an anatase TiO_2_ catalyst on the cathode^16^. The reaction equations can be described as follows.

$${\rm{HOOC}}\mbox{--}{\rm{COOH}}+4{{\rm{H}}}^{+}+4{{\rm{e}}}^{\mbox{--}}\to {\rm{HOOC}}\mbox{--}{{\rm{CH}}}_{2}{\rm{OH}}+{{\rm{H}}}_{2}{\rm{O}}\,({\rm{cathode}})$$

$$2{{\rm{H}}}_{2}{\rm{O}}\to {{\rm{O}}}_{2}+4{{\rm{H}}}^{+}+4{{\rm{e}}}^{-}\,({\rm{anode}})$$

We believe that GC solution can be used as an energy storage chemical because the theoretical capacity of GC solution produced through a four-electron reduction of saturated aqueous solution of OX (≈3.89 M) at 60 °C, which is one of the optimal temperatures for the OX reduction as we discuss later, reaches around 417 Ah l^−1^, which is 190 times higher than that of hydrogen gas (2.2 Ah l^−1^ (SATP)). However, the batch-wise electrolyser, which limits its continuous operation and requires mixing of an electrolyte such as Na_2_SO_4_, is not suitable for the purpose of alcohol production. One idea to solve these disadvantages is to employ a polymer electrolyte electrolyser^17^ because this type enables flow reactions and does not require any electrolytes in reaction solutions due to the existence of solid polymer electrolyte directly contacting with electrode catalysts. So far, various organic compounds have been successfully converted in the liquid phase using this type of polymer electrolyte electrolyser, but their conversions were very low^18–21^. However, there is a lack of reports on alcohol electrosynthesis from carboxylic acid using a polymer electrolyte electrolyser.

Here, we first report the fabrication of a flow-type polymer electrolyser for the continuous production of an alcohol from a carboxylic acid, which is named a polymer electrolyte alcohol electrosynthesis cell (PEAEC, illustrated in the Fig. 1b). A novel membrane-electrode assembly (MEA) for PEAEC is newly developed. The cathode in PEAEC is required to have electron conductivity, substrate diffusivity, and high selectivity for GC production, i.e. selective OX reduction with high overpotential for H_2_ production. We firstly prepared novel cathodes that enable efficient diffusion and conversion of substrates. For this purpose, Ti mesh or Ti felt, to which the reaction solution is permeable, was selected as an electrode material. Rigid linking between electrode and catalysts is also indispensable. Thus, we directly grow porous TiO_2_ on the electrode using Ti ions eluted from the Ti electrodes through a two-step hydrothermal reaction^22^. The MEA for the PEAEC was prepared using the TiO_2_-decorated Ti mesh (TiO_2_/Ti-M) or Ti felt (TiO_2_/Ti-F) electrode as a porous cathode, Nafion 117 as a solid polymer electrolyte, and gas-diffusion carbon electrode loading IrO_2_ (IrO_2_/C) as an anode (for water oxidation). The performance of the prepared electrodes for OX reduction was evaluated and optimized using both a two-compartment electrolyser and the PEAEC by changing various parameters, such as reaction temperature (25–70 °C), applied voltage (1.8–3.0 V), concentration of OX (0.01–1 M), flow rate (0.1–1.0 ml min^−1^), reaction area (1–25 cm^2^), and amount of catalysts. Under the optimal condition, the PEAEC with TiO_2_/Ti-F exhibited a maximum energy conversion efficiency of 49.6% (area: 4 cm^2^, applied voltage: 2.0 V) and almost 100% conversion (99.8%) of 1 M OX (area: 25 cm^2^, applied voltage: 3.0 V), which is an almost saturated solution at room temperature.

## Results and Discussion

### Preparation and characterization of the electrodes

**Figure 2:**
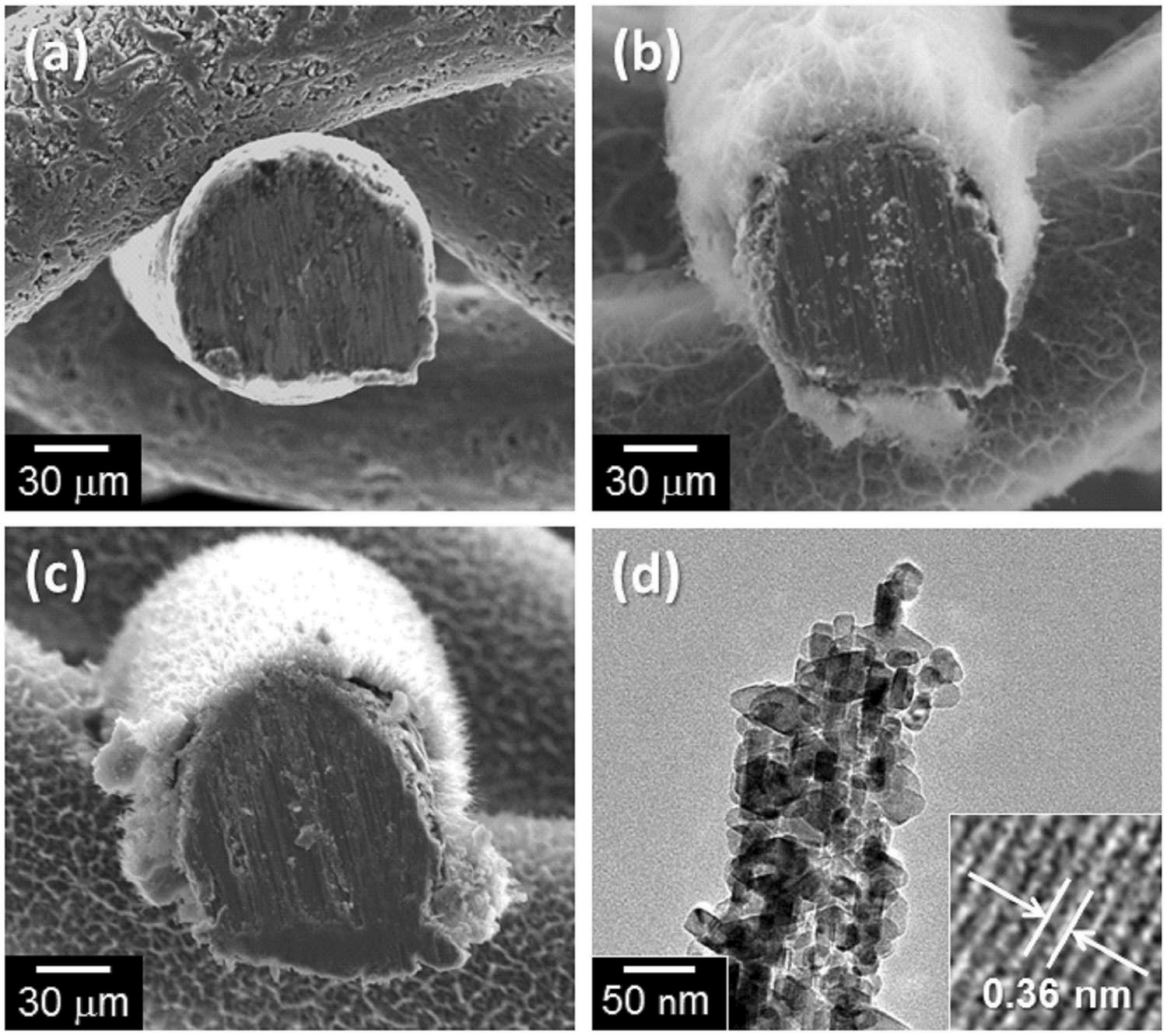
SEM images of (**a**) Ti mesh before the hydrothermal reaction, (**b**) after the first-step reaction taking 12 h (H_2_Ti_2_O_5_·H_2_O on Ti mesh), and (**c**) after the second-step reaction (TiO_2_/Ti-M). (**d**) A TEM image of a TiO_2_ fiber deposited on the TiO_2_/Ti-M after the two-step hydrothermal reaction (first step: 12 h, second step: 24 h). A magnified fringe pattern is shown in the inset.

The cathode was prepared through a growth of anatase TiO_2_ catalyst on Ti mesh (or Ti felt) by employing two-step hydrothermal reactions (Fig. S2)^22^. SEM images of Ti mesh before reaction, after the first-step hydrothermal reaction (12 h), and after the second-step reaction are shown in the Fig. 2a–c. The first step led to the formation of fibrous solids around Ti lines by dissolving Ti metal (Fig. 2b). The X-ray diffraction (XRD) patterns (Fig. S3) indicated that the microfibres formed on the Ti line are a crystalline H_2_Ti_2_O_5_·H_2_O, as is the case with the previous report on Ti foil^22^. In the XRD pattern after the second step, diffraction peaks of anatase TiO_2_ clearly appeared while those from H_2_Ti_2_O_5_·H_2_O (Fig. S3) disappeared, indicating the formation of anatase TiO_2_ through a hydrolysis of the H_2_Ti_2_O_5_·H_2_O. The SEM image of the prepared TiO_2_/Ti-M (Fig. 2c) shows that the formed anatase TiO_2_ also constructs a fibrous structure around the Ti lines with a direct contact with them. TEM observation for the TiO_2_ fibre (Fig. 2d) revealed that the fibre is composed of small TiO_2_ particles with diameters of approximately 20–30 nm with good crystallinity, which can be confirmed from the clear lattice fringes (inset of Fig. 2d). By changing the reaction time for the first step in the range from 1 to 72 h (the second step is fixed at 24 h), we observed a gradual increase of the oxidized species (H_2_Ti_2_O_5_·H_2_O, or TiO_2_ after the second step) as shown in the SEM images (Fig. S4). XRD patterns also suggested the gradual growth of TiO_2_ species (after the second step) depending on the reaction time in the first step (Fig. S5). The amount of TiO_2_ loaded on the Ti mesh linearly varied with the reaction time in the first step (Fig. S6). These results clearly indicate that the loading amount of the anatase TiO_2_ catalysts can be easily controlled just by changing the reaction time for the hydrothermal reaction in the first step.

To clarify the effect of the hydrothermal reaction time on catalytic performance for the electrochemical reduction of OX, we performed chronoamperometry using a two-compartment electrolyser with a three electrode system. Figure S7 shows OX conversion and Faradaic efficiency after the chronoamperometry for 2 h at an applied potential of 0.76 V (vs. RHE) using each TiO_2_/Ti-M electrode. The conversion of OX increased with increasing the hydrothermal reaction time below 12 h, indicating that the amount of TiO_2_ catalyst on the Ti line is important to accelerate the catalytic reduction of OX in this region. Faradaic efficiency for the target product (GC) also drastically increased with increasing the hydrothermal reaction time, suggesting that the metallic Ti surface, uncovered by TiO_2_, preferably generates hydrogen gas as a by-product. Above 12 h, the conversion of OX and Faradaic efficiency are almost independent of the hydrothermal reaction time in the first step, indicating that the extra amount of TiO_2_ on the electrode does not contribute to increasing catalytic performance. This might be because TiO_2_ crystals located far from the metal Ti electrode do not work as electrocatalysts due to high electrical resistivity. Note that the excess amount of TiO_2_ (e.g. 24 h of the first step) is not preferable for fabrication of MEA because it causes a peel-off of the cathode electrode from Nafion after a hot press. Thus, we employed 12 h as an optimal hydrothermal reaction time in the first step for fabrication of the TiO_2_/Ti-M electrode for the PEAEC.

To check the optimum temperature for the catalysis with the prepared TiO_2_/Ti-M electrode (12 h for the first-step hydrothermal reaction), we conducted linear sweep voltammetry (LSV) at 25, 50, 60, and 70 °C (Fig. S8) using an aqueous solution of Na_2_SO_4_ with and without OX substrate. The TiO_2_/Ti-M cathode showed quite different LSV curves in the presence or absence of OX substrate around 50–60 °C, which clearly indicates that OX reduction preferably occurs on the anatase TiO_2_ catalyst rather than the hydrogen evolution reaction in this temperature region, which is similar to our previous report regarding TiO_2_/Ti foil electrode^16,23^. Onset potentials are listed in Table S1. There is almost no difference in the onset potentials at 70 °C, indicating that the overpotential for the hydrogen evolution is very low above 70 °C, which is not desirable for the selective reduction of OX.

To confirm the applicability of this method to another Ti electrode to obtain a higher surface area, we applied the optimized procedure to construct a cathode using the Ti felt, where thinner Ti lines are densely folded. Figure S9 shows photographs and SEM images before and after the optimized two-step hydrothermal treatment (first step: 12 h, second step: 24 h). We could obtain a grey-coloured TiO_2_/Ti-F electrode, as in the case with the TiO_2_/Ti-M electrode (Figure S9a–b). TiO_2_ microcrystals were also formed on the surface of Ti lines of Ti felt (Fig. S9d). Figure S10 shows nitrogen adsorption isotherms of TiO_2_/Ti-M and TiO_2_/Ti-F electrodes, measured at 77 K. BET surface areas of TiO_2_/Ti-M and TiO_2_/Ti-F were estimated to be 2.4 and 19.6 m^2^ g^−1^, respectively, showing that TiO_2_/Ti-F has almost 10 times larger surface area. XRD patterns of the prepared TiO_2_/Ti-F are shown in Fig. S11. Similarly to observations on TiO_2_/Ti-M, deposition of H_2_Ti_2_O_5_·H_2_O and TiO_2_ was recognized after the first step and the second step, respectively. These results clearly show that this method is generally applicable for the fabrication of TiO_2_ catalysts on the surface of Ti electrodes.

We also measured LSV curves of the prepared IrO_2_/C anode. The current gained at the same applied potential varied depending on the temperature, but saturated above 60 °C (Fig. S12). According to the results of the TiO_2_/Ti-M cathode, 60 °C seems to be one of the optimal temperatures for the electrosynthesis of GC using a pair of redox reactions of OX reduction and water oxidation on the TiO_2_/Ti-M cathode and the IrO_2_/C anode.

### Electrosynthesis of GC using the flow-type PEAEC

**Figure 3:**
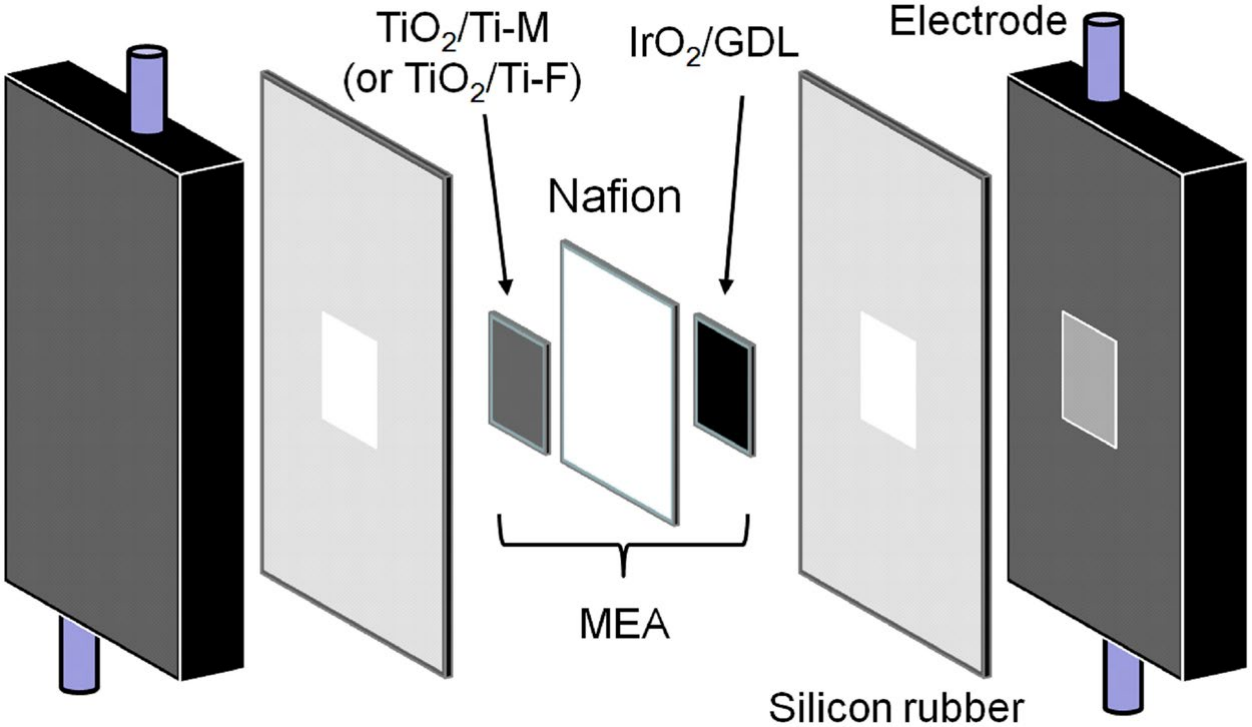
Assembly of building blocks for the PEAEC.

**Figure 4:**
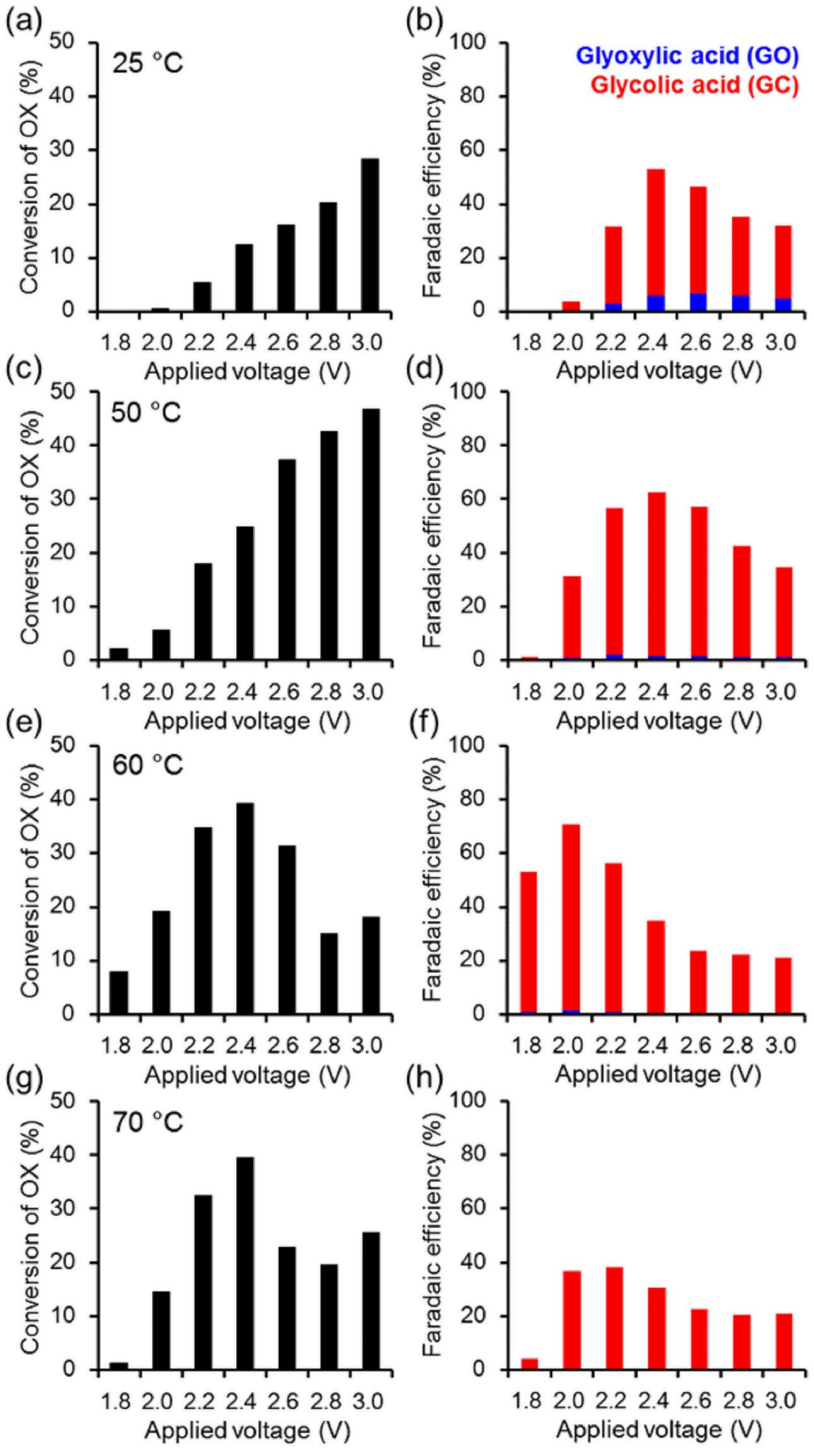
Performances of oxalic acid reduction through the PEAEC with TiO_2_/Ti-M cathode operating with a reaction area of 4 cm^2^, flow rate of 0.5 ml min^−1^, and OX concentration of 0.03 M. Conversions of OX at (**a**) 25, (**c**) 50, (**e**) 60, and (**g**) 70 °C and Faradaic efficiency at (**b**) 25, (**d**) 50, (**f**) 60, and (**h**) 70 °C at each applied voltage. Red and blue colors correspond to GC and GO, respectively.

The prepared electrodes were successfully combined with a Nafion membrane through a hot press method to obtain an MEA. The PEAEC was constructed by attaching the MEA between carbon electrodes having flow channels (Fig. 3). To evaluate the performance of the prepared PEAEC for the continuous electrochemical production of GC from OX, we first conducted controlled-potential electrolysis of OX (0.03 M aqueous solution with no additional electrolyte) at various voltages in the range from 1.8 to 3.0 V using an MEA with the TiO_2_/Ti-M cathode at 25 °C (reaction area: 4 cm^2^, flow rate: 0.5 ml min^–1^). Figure S13 shows a high-performance liquid chromatograph (HPLC) chart of the downstream solution of the cathode side before and after applying the voltage of 2.4 V. The peak of OX was decreased by applying the voltage while the peak of the target product GC increased, showing that the continuous electrochemical production of GC from OX was successfully achieved by using the PEAEC without any additional electrolyte in the reaction solution. A slight amount of GO was also observed as an intermediate product. Note that the carbon balance of the products was observed as almost one (Fig. S14), confirming that the carbon products (GC and GO) were derived from the catalytic reduction of OX. Figure 4 shows the temperature dependence of the OX conversion and Faradaic efficiency at each applied voltage. Both OX conversion and Faradaic efficiency for the carbon products completely depend on the reaction temperature. OX conversion at a lower applied voltage (below 2.4 V) significantly increased with increasing temperature, suggesting that the overpotential for the OX reduction decreases with increasing temperature. On the other hand, Faradaic efficiency decreases, especially above 70 °C, suggesting the increase of hydrogen production, which is ascribed to lowering of overpotential for the hydrogen production above 70 °C. These observations are almost consistent with the results of the LSV measurements. Thus, we could recognize that the optimal temperature for PEAEC operation is around 60 °C. In these experimental conditions (reaction area: 4 cm^2^, flow rate: 0.5 ml min^–1^, 0.03 M OX), the maximum Faradaic efficiency for GC achieved 69.4% at 2.0 V. The theoretical electrolysis voltage for the PEAEC producing GC from OX (*E*_GC_) is 1.1 V, calculated from the standard redox potentials of GC production from OX (HOOC–COOH + 4 H^+^  + 4e^−^ → HOOC–CH_2_OH + H_2_O, 0.13 V vs. SHE) and water oxidation (2H_2_O → O_2_ + 4 H^+^ + 4e^−^, 1.23 V vs. SHE). According to this value, the efficiency of energy storage in GC (*η*_GC_) can be calculated to be 38.1%, using the following equation

1 $${\eta }_{GC}=\frac{{E}_{GC}\times {F}_{GC}}{{E}_{appl}}$$

where *E*_appl_ is the applied voltage, and *F*_GC_ is the Faradaic efficiency for GC production. In this condition, the conversion of OX did not reach 100% but was 39.4% at 2.4 V even at the optimal temperature of 60 °C. It is also clear that production of an intermediate product, GO, becomes almost zero above 60 °C, while a considerable amount of GO (≈7% FE) was observed at 25 °C. Figure S15 shows the average current density of the PEAEC at each temperature. The current density increased with increasing temperature below 2.6 V and reached 23 mA cm^−2^ at 2.6 V at 60 °C. Above 60 °C, the current density decreased with increasing applied voltage, which might be because of degradation of the anode due to carbon corrosion.

To achieve maximum performance of the PEAEC at 60 °C, we changed various factors, such as reaction area (1–25 cm^2^), flow rate (0.1–2 ml min^−1^), and OX concentration (0.01–1 M). Figure S16 shows the dependence of OX conversions on these factors. The OX conversion monotonically decreased with increasing flow rate and OX concentration, but increased with the electrode area, which clearly suggests that the reaction rate on the cathode is not high enough to achieve 100% conversion of OX. It should be noted that the Faradaic efficiency for GC increases with increasing OX concentration of the reaction solution, indicating that the selective reduction of OX, rather than hydrogen evolution, preferably occurs in OX solution with higher concentrations, e.g. 1 M solution. From these results, we realized that higher OX conversion with higher Faradaic efficiency for GC production using the PEAEC would be achieved by applying a large reaction area, low flow rate, and high concentration of OX solution. The amount of IrO_2_ on the anode was also varied for optimization. Figure S17 shows average current density, OX conversion, and Faradaic efficiency of the PEAEC with various amounts of IrO_2_ catalyst (1–10 mg cm^−2^). The maximum current density at 2.6 V monotonically increased with increasing the amount of IrO_2_ catalysts (in the range from 23 to 112 mA cm^−2^), indicating that the anodic reaction is the rate-determining step of the PEAEC operation, which can be ascribed to the high overpotential for the water oxidation. Note that the PEAEC with 10 mg cm^−2^ of IrO_2_ did not show significant degradation above 2.6 V, which might be because of minimization of exposure of the carbon electrode surface to the reaction solution. It should also be noted that 10 mg cm^−2^ seemed to be the maximum amount of catalyst in our hand painting method because of a peel-off of anode paper from the MEA after a hot press. Thus, we employ 10 mg cm^−2^ as an optimal amount for the PEAEC.

The difference in performance between the TiO_2_/Ti-M and TiO_2_/Ti-F cathodes was tested using the PEAEC. Figure S18 shows a comparison of the performance between TiO_2_/Ti-M and TiO_2_/Ti-F cathode in the PEAEC (OX concentration: 1 M, reaction area: 4 cm^2^, flow rate: 0.5 ml min^−1^, IrO_2_: 10 mg cm^−2^, temperature: 60 °C). It is clear that the TiO_2_/Ti-F shows higher OX conversion (26.6% at 3.0 V) than TiO_2_/Ti-M (17.1% at 3.0 V), indicating that the reaction rate on the TiO_2_/Ti-F cathode is faster than that on TiO_2_/Ti-M. This might be ascribed to the higher surface area of TiO_2_/Ti-F (19.6 m^2^ g^−1^) than that of TiO_2_/Ti-M (2.4 m^2^ g^−1^). Surprisingly, the Faradaic efficiency of TiO_2_/Ti-F for GC is higher than that of TiO_2_/Ti-M at all applied voltages and achieves 90.2% at 2.0 V at maximum, which leads to 49.6% for the efficiency of energy storage in GC as a maximum. This shows that the hydrogen production is significantly suppressed by applying the TiO_2_/Ti-F electrode as the cathode. We believe that one reason for this high selectivity for GC is derived from high TiO_2_ coverage of Ti felt because exposed metal Ti preferably produces hydrogen. Thus, we employed TiO_2_/Ti-F as an optimized cathode.

**Figure 5:**
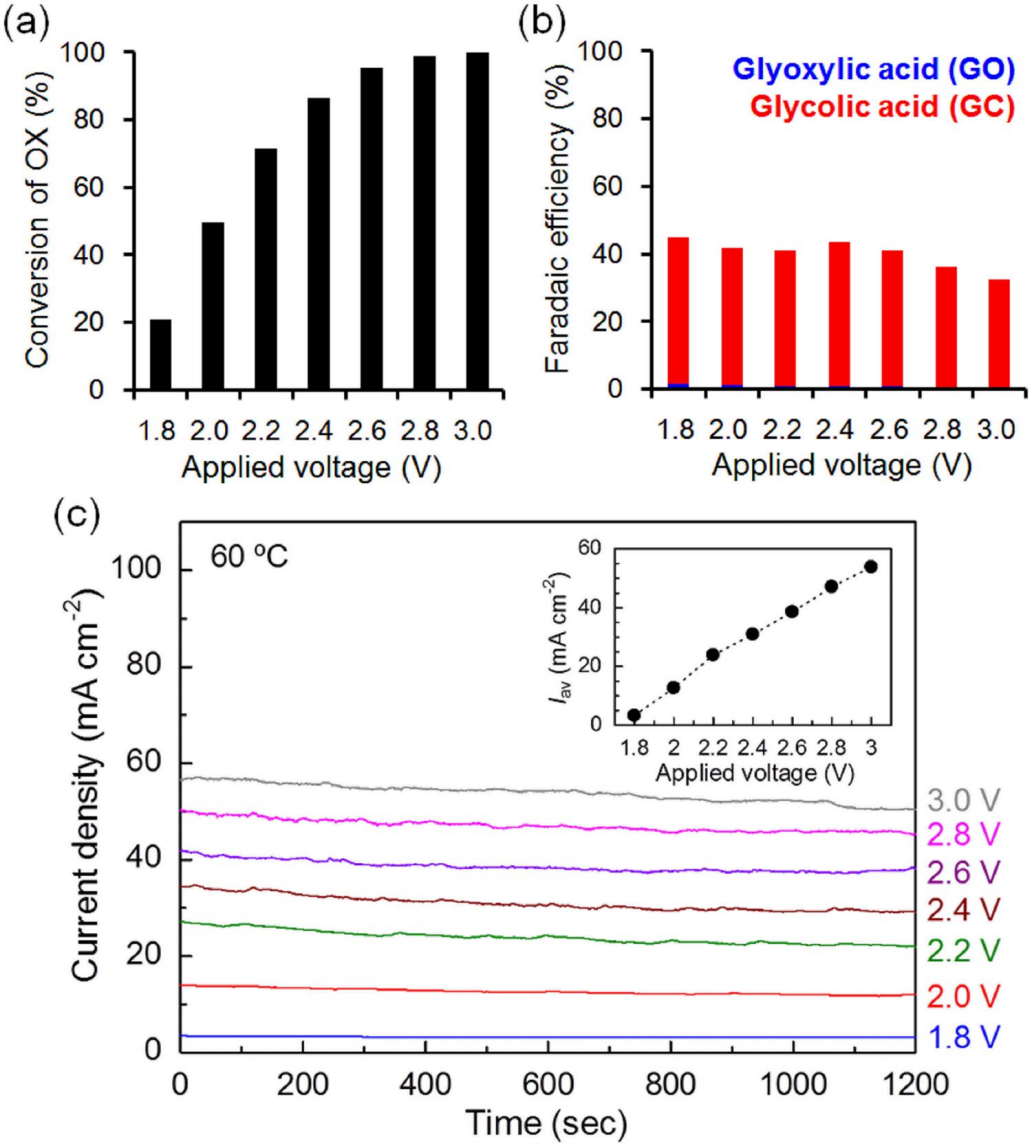
(**a**) OX conversions, (**b**) Faradaic efficiency, and (**c**) current density during the operation of the PEAEC equipped with TiO_2_/Ti-F cathode (OX concentration: 1 M, reaction area: 25 cm^2^, flow rate: 0.1 ml min^–1^, IrO_2_: 10 mg cm^–2^, temperature: 60 °C). Inset shows averaged current density (*I*_av_) at each applied voltage.

According to the above experiments, we constructed the PEAEC with TiO_2_/Ti-F cathode to achieve complete conversion of OX by employing the following optimized conditions: OX concentration = 1 M, reaction area = 25 cm^2^, flow rate = 0.1 ml min^–1^, IrO_2_ = 10 mg cm^−2^, and temperature = 60 °C. Figure 5 shows OX conversion, Faradaic efficiency, and current density of the PEAEC. Surprisingly, OX conversion finally reached almost 100% (99.8% at 3.0 V applied voltage) with moderate Faradaic efficiency (31.9% for GC production at 3.0 V), while we used 1 M OX solution, which is an almost saturated solution at room temperature (solubility of OX at 20 °C is around 1.01 M)^24^. Note that the Faradaic efficiency for GO production was vanishingly small (0.4% at 3.0 V). *η*_GC_ is calculated to be 26.5% at maximum (*E*_appl_ = 1.8 V, *F*_GC_ = 43.4%) in this case. Lowering the applied voltage and increasing the Faraday efficiency would be important for further increase in the efficiency for energy storage. Figure 5c shows the current density of the PEAEC. The maximum average current density was 53.8 mA cm^−2^ at 3.0 V. The power density for energy storage in GC (*P*_GC_) can be expressed with the equation,

2 $${P}_{GC}={E}_{GC}\times I\times {F}_{GC}$$

in which *I* is the current density of the PEAEC. Considering that the *I* and *F*_GC_ at 3.0 V are 53.8 mA cm^−2^ and 31.9%, respectively, the power density for the energy storage in GC is calculated to be 18.9 mW cm^−2^ at maximum. Water electrolysers^5,17^, which continuously produce hydrogen as an energy carrier, and redox flow batteries^25,26^, which continuously convert electrical energy into chemical energy of catholyte (and anolyte) stored outside the cell, are some of the competitive devices for the PEAEC. The current density of our PEAEC (<60 mA cm^−2^) is approximately one order lower than that of commercial water electrolysers (250–1000 mA cm^−2^)^5^. Considering that GC solution is storable in a common container under ambient conditions without liquefaction at low temperatures or under high pressures, GC has a significant advantage as an energy carrier compared with chemically active and gaseous hydrogen. Furthermore, if we could recognize the OX solution as a flowable electron pool for the energy storage, the theoretical volumetric capacity of the flowed 1 M OX solution can be calculated as 107 Ah l^−1^ (four-electron reduction of OX to form GC). This value is approximately 50 times higher than that of hydrogen gas (2.2 Ah l^−1^ (SATP)) and almost double that of vanadium-based catholyte (60 Ah l^−1^)^25^, implying that conversion of the carboxylic acid group into a hydroxyl group (alcohol) with the PEAEC has great potential as a flow-type energy storage device. It should be noted that the GC can generate electricity on a fuel cell accompanied by the production of OX, as we previously reported^16^. In addition, as noted above, the theoretical capacity of GC solution produced through a four-electron reduction of saturated aqueous solution of OX at the operation temperature (≈3.89 M at 60 °C) is around 417 Ah l^−1^, which is one order higher than that of a typical redox flow battery and 190 times higher than that of hydrogen. Thus, we believe that the PEAEC producing alcohols would be a significant device for energy storage, while some of the issues such as the low energy efficiency (our PEAEC < 27%, redox flow battery ≈80%^26^), i.e. low Faradaic efficiency for GC, and high overpotential should be solved in the future.

In summary, we succeeded in the construction of a novel flow-type PEAEC continuously converting OX into GC without any addition of electrolyte, by employing TiO_2_/Ti-M or TiO_2_/Ti-F electrodes as cathode materials. Porous anatase TiO_2_ catalyst was successfully loaded on Ti mesh or Ti felt, having substrate diffusivity, through a two-step hydrothermal reaction. We demonstrated that the PEAEC continuously converted 1 M OX solution, having a volumetric capacity of 107 Ah l^−1^, into GC with almost 100% conversion (99.8%). Maximum efficiency of energy storage in GC using the PEAEC reached 49.6%. We believe that alcohol production using the PEAEC can be a new candidate as a flow-type energy storage device and that novel ideas for the construction of the electrode materials for the PEAEC will attract much interest in materials science.

## Methods

### Materials

Ti mesh (100 mesh, diameter of Ti line is 100 μm, twill weave) and Ti felt (WB/Ti/20/150, the diameter of the Ti line is approximately 20 μm, the density of Ti is 150 g m^–2^) were purchased from Manabe Industry, Co. and Nikko Techno, Co., respectively. IrO_2_ (Wako Chemical), NaOH (Kanto Chemical), and OX·2H_2_O (Kanto Chemical) were used as purchased. Nafion 117 (0.007 inch thickness), Nafion NRE-212 (0.002 inch thickness), and Nafion solution (5 wt%) were purchased from Sigma-Aldrich, Co. Carbon paper, SIGRACET GDL 25BC, was purchased from SGL group. Carbon felt, E-525, was purchased from Kureha, Co. A home-designed two-compartment electrolyser was used. Fuel cell evaluation systems (carbon block electrodes with flow channels, 1–25 cm^2^, serpentine flow) were purchased from ElectroChem, Inc. and used for the PEAEC.

### Preparation of electrodes

Ti mesh (area: 1–25 cm^2^) was put in a Teflon-lined autoclave with 30 ml of 1 M NaOH aqueous solution. The autoclave was then heated to 220 °C for various reaction times (1–72 h) to optimize reaction conditions to grow H_2_Ti_2_O_5_·H_2_O on the electrode as the first step. After that, the Ti mesh was washed with water and immersed in 0.1 M HCl aqueous solution for 10 min followed by washing with water and ethanol and drying under air. As the second step, the treated Ti mesh was put in a Teflon-lined autoclave again with 40 ml of water and kept at 200 °C for 24 h to convert H_2_Ti_2_O_5_·H_2_O into anatase TiO_2_ on the electrode. After washing with water and ethanol, the electrode was then dried under air. The colour of the electrode changed to light grey (H_2_Ti_2_O_5_·H_2_O/Ti-M or TiO_2_/Ti-M) from dark grey (Ti mesh) due to the deposition of H_2_Ti_2_O_5_·H_2_O after the first step or TiO_2_ after the second step on the Ti mesh (Fig. S1). An optimized condition (12 h for the first step) was also applied to the preparation of the Ti felt electrode to obtain a highly porous TiO_2_/Ti-F electrode. An anode electrode was prepared the by hand painting method^27,28^ with a gas-diffusion carbon electrode (GDL, Sigracet 25 BC). A typical procedure for preparation of catalyst ink was that a mixture containing 20 mg of ground IrO_2_ powder, 120 μl of Nafion solution (5 wt%), 1.2 ml of 2-propanol, and 1.2 ml of water was sonicated for several tens of minutes. The ink was then painted on the GDL having an area of 20 cm^2^ (IrO_2_: 1 mg cm^−2^). The anode was cut into a target area (e.g. 2 × 2 cm^2^) with a square shape before use. When we changed the amount of anode catalyst (e.g. 10 mg cm^−2^), the amount of the loaded ink was just changed with the same composition of the ink.

### Characterizations

XRD patterns of TiO_2_/Ti-M were collected using a Rigaku SmartLab diffractometer equipped with a Cu-*Kα* X-ray source (*λ* = 1.5418 Å). Scanning electron microscope (SEM) images and transmission electron microscope (TEM) images were taken on a JSM-IT100 (JEOL, Co.) at an accelerating voltage of 20 kV and JEM-2100 (JEOL, Co.) at an accelerating voltage of 200 kV, respectively. Adsorption isotherms of N_2_ were measured at 77 K using BEL-SORP max (Microtrac BEL, Co.).

For the evaluation of the performance of TiO_2_/Ti-M electrodes, LSV was conducted using a three-electrode system containing the prepared TiO_2_/Ti-M as a working electrode, Ag/AgCl as the reference electrode, and Pt mesh as the counter electrode with a scan rate of 10 mV s^−1^, after bubbling Ar for 15 min. An aqueous solution of Na_2_SO_4_ (0.2 M) with OX (0.03 M) was used after adjusting the pH to 1.6 by adding dilute H_2_SO_4_ for the measurement. LSV in the absence of the substrate (OX) was also measured using blank Na_2_SO_4_ aqueous solution (pH was adjusted to 1.6 by adding dilute H_2_SO_4_.). For LSV measurements for IrO_2_/C anode, blank Na_2_SO_4_ (0.2 M) aqueous solution was used (pH = 6.4). Chronoamperometry using the TiO_2_/Ti-M cathodes prepared with various reaction times of the first step (1–72 h) was performed using a three-electrode system (reference electrode: Ag/AgCl, counter electrode: Pt coil) with a two-compartment electrolyser. 40 ml of reaction solution (aqueous solution of Na_2_SO_4_ (0.2 M) with OX (0.03 M), pH = 2.1) was put in one side including working and reference electrodes; 40 ml of blank Na_2_SO_4_ (0.2 M) aqueous solution (pH was fixed to 2.1 by addition of H_2_SO_4_) was put in the other side containing the counter electrode. The two solutions were separated by Nafion NRE-212.

### Fabrication of the PEAEC

The MEA was prepared by a hot press method. Note that we did not use any ionomers for the preparation of the cathode because it lowered the performance of the PEAEC. The prepared TiO_2_/Ti-M or TiO_2_/Ti-F cathode, Nafion, and IrO_2_/C anode were pressed and kept at 120 °C for 4 minutes. After cooling to room temperature, the MEA was extracted from the pressing machine. The PEAEC was constructed using the MEA containing TiO_2_/Ti-M or TiO_2_/Ti-F cathode, Nafion, and IrO_2_/C anode. The MEA was set between carbon block electrodes with serpentine flow patterns (reaction area is 1–25 cm^2^), which are commercially available fuel cell evaluation systems (ElectroChem Inc.). Silicon rubber was used as a gasket. Calcined Ti mesh and carbon felt were also inserted behind the MEA on the cathode and anode sides, respectively, to make a better electrical contact with the carbon block electrode.

### Electrolysis using the flow-type PEAEC

A typical procedure for the electrolysis is shown below. As reaction solutions for cathode and anode, 0.03 M aqueous solution of OX (pH = 1.6) and pure water were used, respectively. To avoid the influence of the O_2_ reduction reaction during the electrolysis, the solution was bubbled once with Ar and degassed by a degasser (Gastorr BG-34, FLOM Co.) before flowing. The reaction solution was continuously flowed using a flow controller (PCS Pump SP-21, LAB-SYSTEM) on each side with a flow rate of 0.5 ml min^–1^. The cell was then heated up to a target temperature (e.g. 60 °C) by a heater attached to the cell. The current was monitored under application of a constant voltage in the range from 1.8 to 3.0 V between the electrodes using a potentiogalvanostat (1280 C, Solartron). After waiting for 3 min for a steady state, the product solution from the cathode was collected for 20 min under each applied voltage. The composition of the product was analysed using an HPLC equipped with refractive index detector (Prominence, Shimadzu Co.) and a separating column for organic acids (KC-811, Shodex Co.). The Faradaic efficiency for each product was determined as follows,

3 $${\rm{Faradaic}}\,{\rm{efficiency}}\,( \% )=\frac{z\times n\times F}{{Q}}\times 100$$

where *z* is the number of electrons to produce a specific product (for GC, z = 4), *n* (mol) is the amount of the specific product, *F* is Faraday’s constant (=96485 C mol^−1^), and *Q* (C) is the charge passed during the electrolysis. Candidates as products of the OX reduction reaction are shown in Fig. S2 ^29^. In this study, we only observed GC as a monovalent alcohol (four electron reductant) and glyoxylic acid (GO) as a monovalent aldehyde (two electron reductant). For optimization of the catalytic performances on the PEAEC, we conducted controlled-potential electrolysis by changing various conditions, e.g., temperature (25–70 °C), OX concentration (0.01–1 M), flow rate (0.1–1.0 ml min^−1^), reaction area (1–25 cm^2^), amount of IrO_2_ catalyst (1–10 mg cm^−2^), and cathode electrode (TiO_2_/Ti-M or TiO_2_/Ti-F).

## Electronic supplementary material

Supplementary Information
