# Supplementary material for: Electrochemical Production of Glycolic Acid from Oxalic Acid Using a Polymer Electrolyte Alcohol Electrosynthesis Cell Containing a Porous TiO2 Catalyst
Source: Sci Rep. 2017 Dec 12;7:17032. doi: 10.1038/s41598-017-17036-3 (PMC5727030; doi:10.1038/s41598-017-17036-3)
Supplement: Supplementary file 1 — Supplementary Information [file 41598_2017_17036_MOESM1_ESM.pdf]

Supplementary Information

Electrochemical Production of Glycolic Acid from Oxalic Acid Using a Polymer Electrolyte Alcohol Electrosynthesis Cell Containing a Porous TiO_2 Catalyst

Masaaki Sadakiyo,*[†,‡] Shinichi Hata,[†] Xuedong Cui,[†] Miho Yamauchi*[†,‡]

[†] International Institute for Carbon-Neutral Energy Research (WPI-I2CNER), Kyushu University, Moto-oka 744, Nishi-ku, Fukuoka, 819-0395, Japan.

[‡] Department of Chemistry, Faculty of Science, Kyushu University, 744 Moto-oka, Nishi-ku Fukuoka 819-0395, Japan.

E-mail: sadakiyo@i2cner.kyushu-u.ac.jp; yamauchi@i2cner.kyushu-u.ac.jp

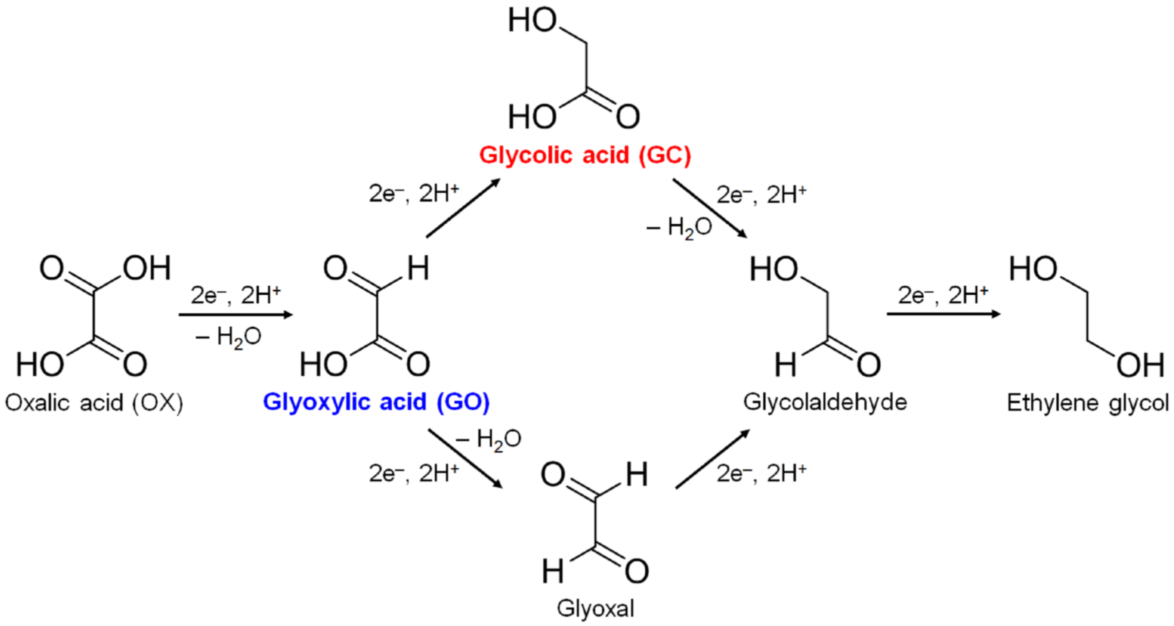

Figure S1. A schematic view of the expected reduced products of OX.

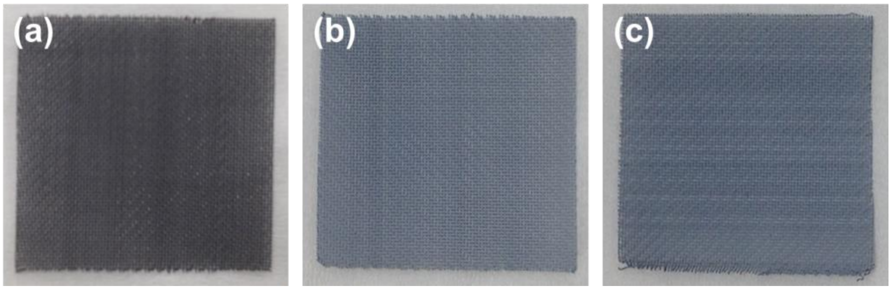

Figure S2. Photographs of (a) Ti mesh before reaction, (b) after the first-step reaction (12 h) ($H_2Ti_2O_5 \cdot H_2O$ on Ti mesh), and (c) after the second-step reaction (24 h) (TiO_2/Ti-M).

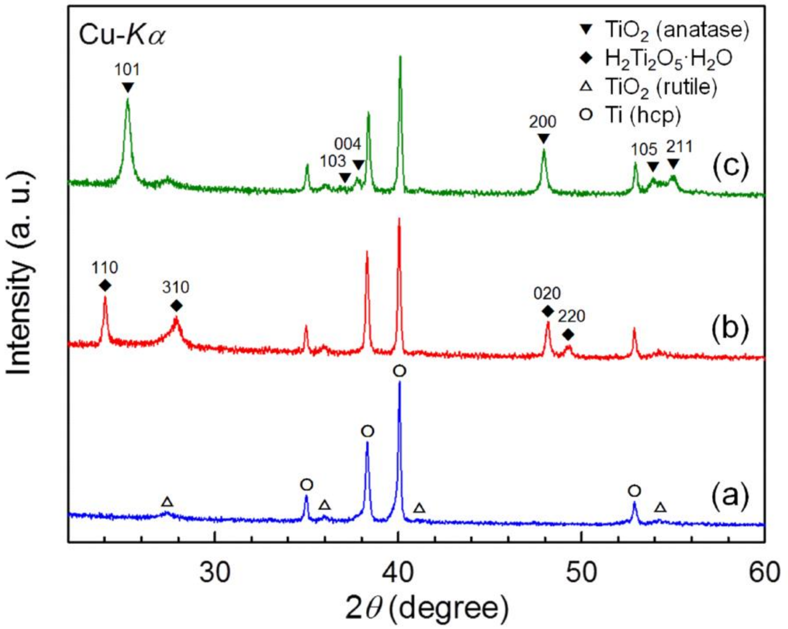

Figure S3. XRD patterns of (a) Ti mesh before reaction, (b) after the first-step reaction (12 h) ($H_2Ti_2O_5 \cdot H_2O$ on Ti mesh), and (c) after the second-step reaction (TiO_2/Ti-M, first step: 12 h, second step: 24 h).

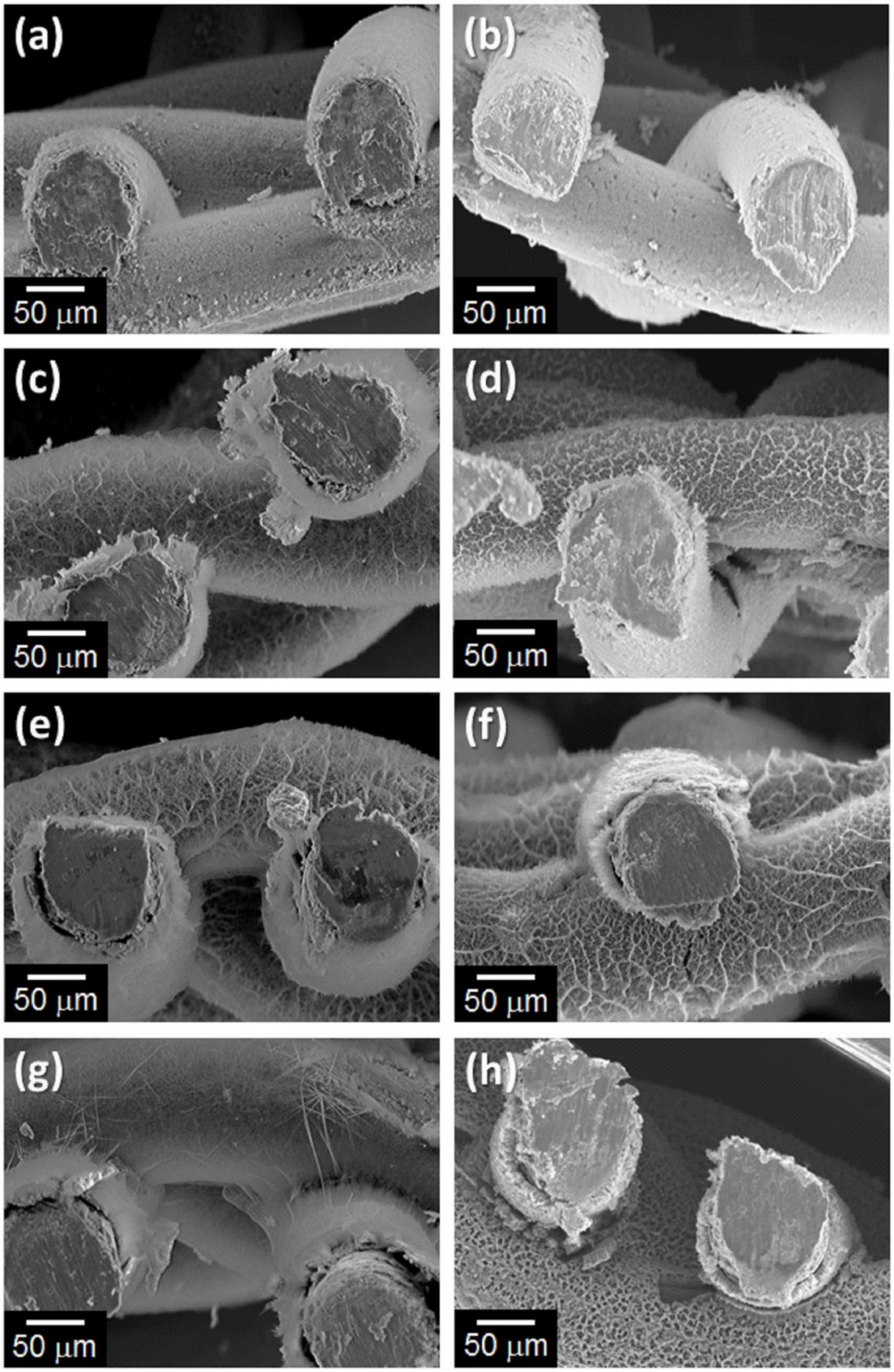

Figure S4. SEM images of Ti mesh after the first step reaction for (a) 6, (c) 12, (e) 24, and (g) 48 h. SEM images of Ti mesh after the second step reaction for 24 h after the first step reaction for (b) 6, (d) 12, (f) 24, and (h) 48 h.

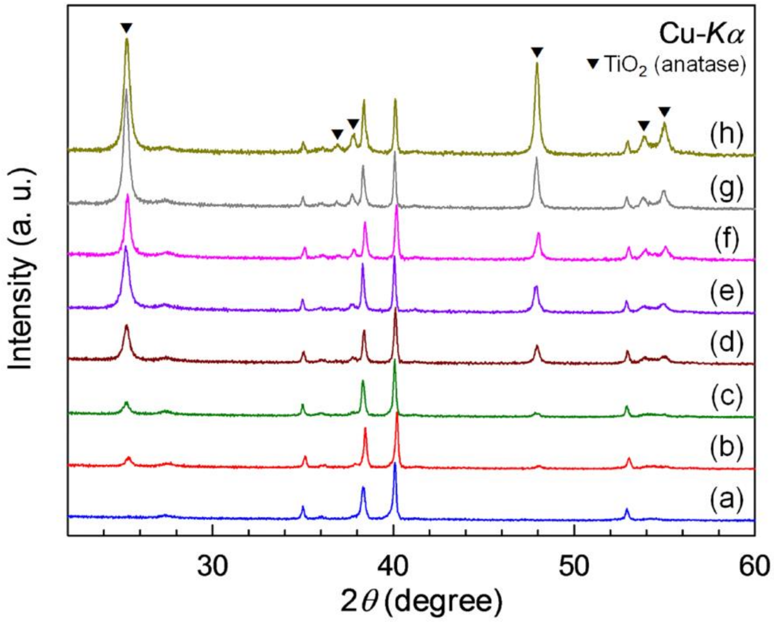

Figure S5. XRD patterns of (a) Ti mesh and TiO_2/Ti-M with the first step hydrothermal reaction time for (b) 3, (c) 6, (d) 12, (e) 18, (f) 24, (g) 48, and (h) 72 h (second step is fixed to 24 h).

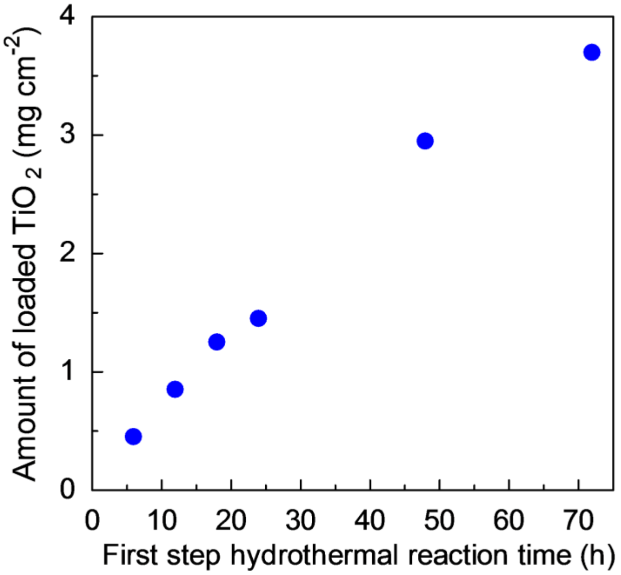

Figure S6. Dependence of the amounts of loaded TiO_2 on the first step hydrothermal reaction time (after the second step (24 h)).

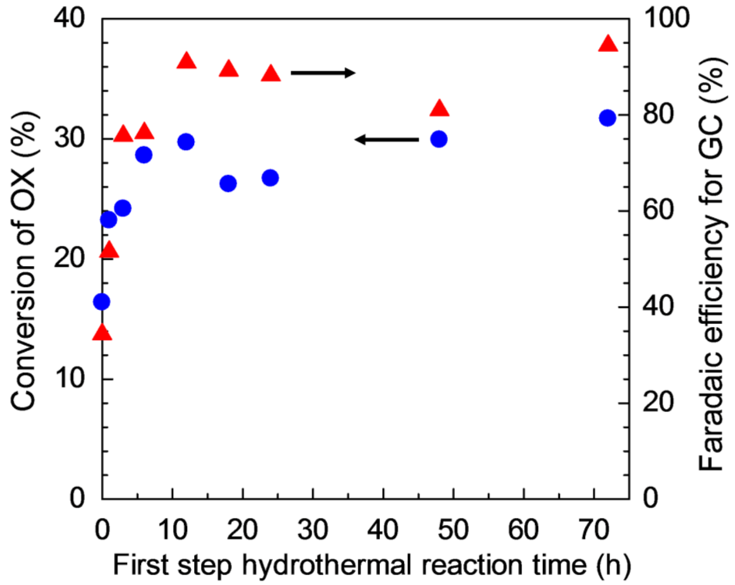

Figure S7. Dependence of (blue) OX conversion and (red) Faradaic efficiency on the first step hydrothermal reaction time, after the chronoamperometry at 0.76 V (vs. RHE) for 2 h using the TiO_2/Ti-M electrode.

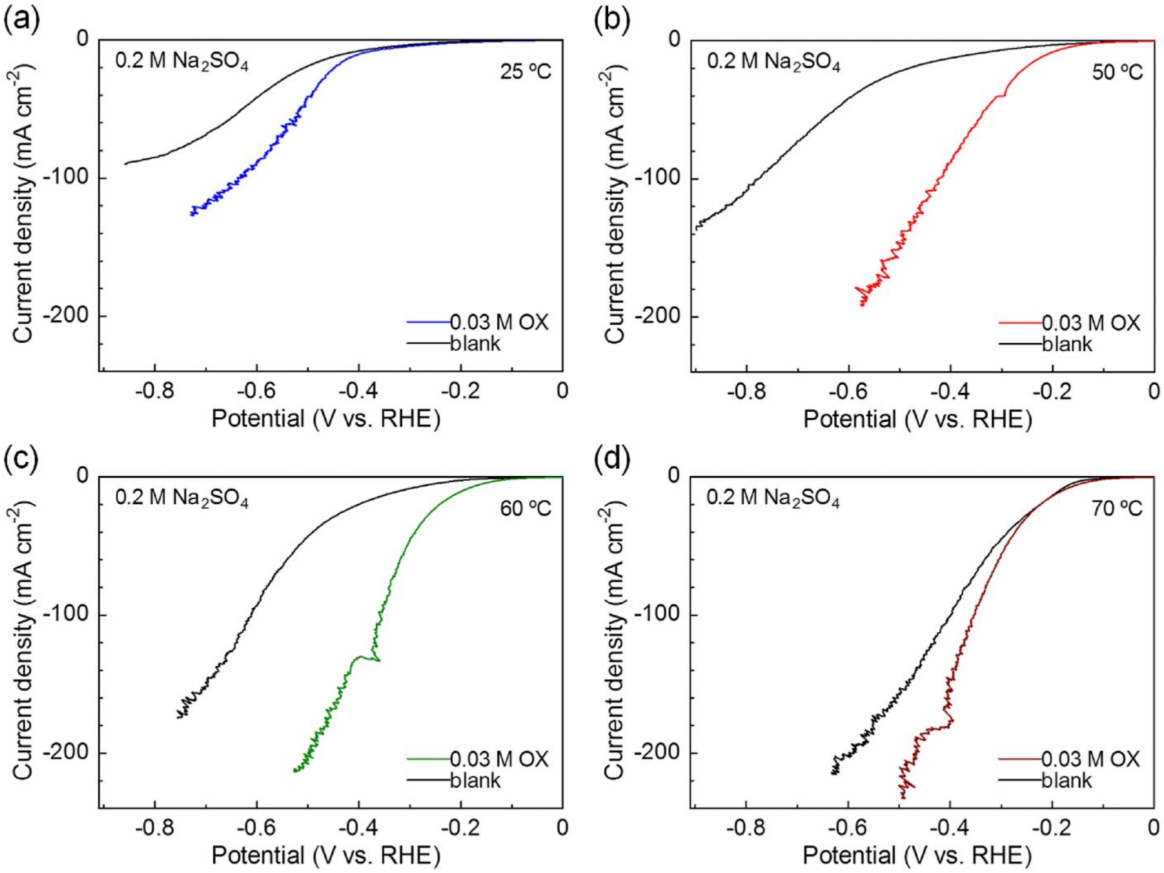

Figure S8. LSV curves of the TiO_2/Ti-M (the first step reaction: 12 h) cathode at (a) 25, (b) 50, (c) 60, and (d) 70 °C.

Table S1. List of onset potentials of the TiO_2/Ti-M (the first step reaction: 12 h) cathode at various temperatures, estimated from LSV curves (potentials at –10 mA reductive current).

	25 °C	50 °C	60 °C	70 °C
Onset potentials of blank (vs. RHE)	–0.428	–0.361	–0.320	–0.187
Onset potentials of 0.03 M OX (vs. RHE)	–0.399	–0.212	–0.197	–0.182

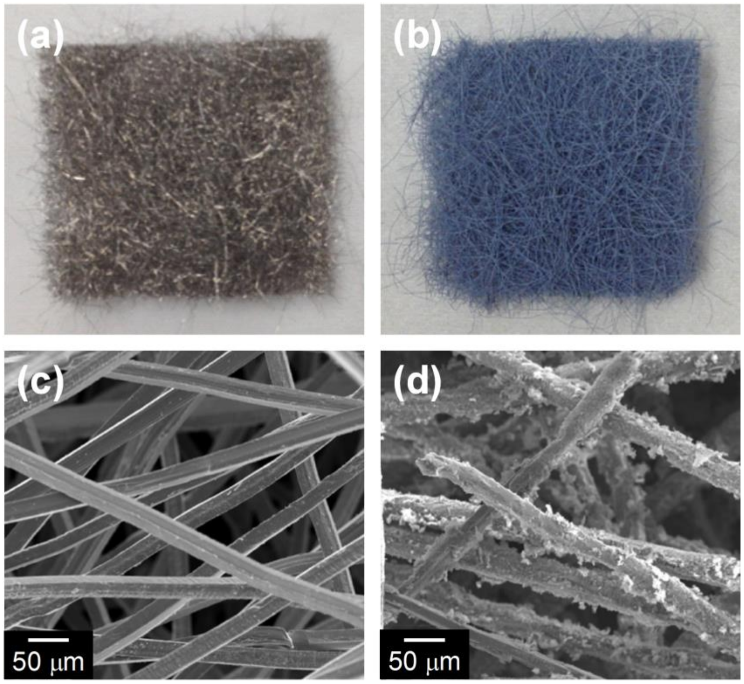

Figure S9. Photographs of (a) Ti felt and (b) TiO_2/Ti-F. SEM images of (c) Ti felt and (d) TiO_2/Ti-F.

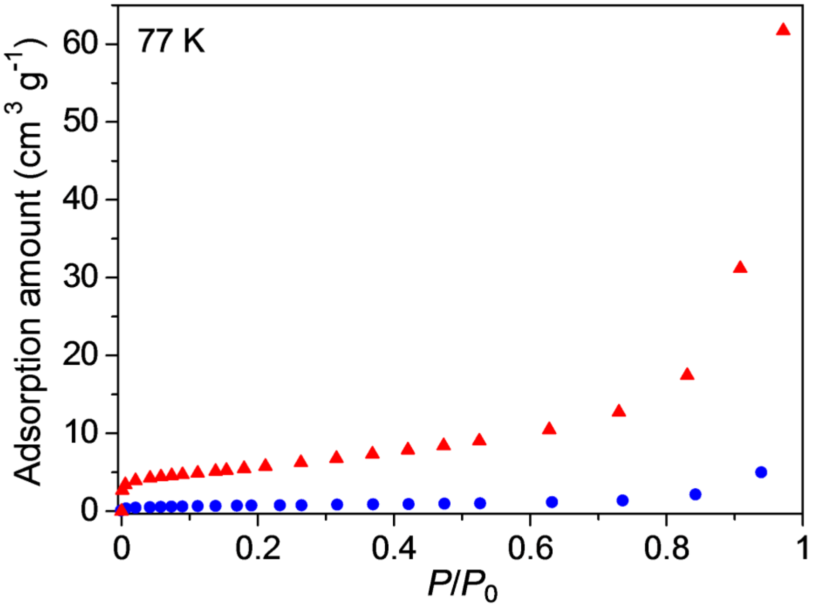

Figure S10. Nitrogen adsorption isotherms of (blue) TiO_2/Ti-M and (red) TiO_2/Ti-F at 77 K.

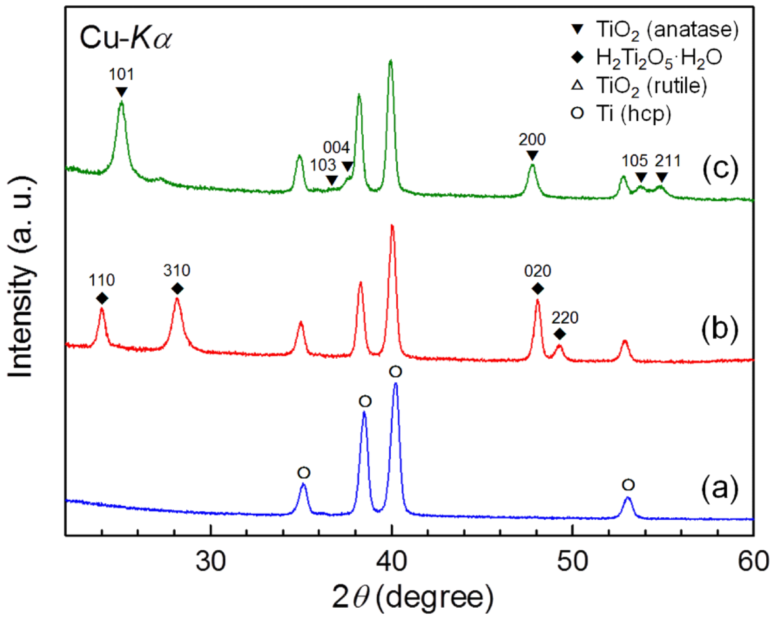

Figure S11. XRD patterns of (a) Ti felt before reaction, (b) after the first-step reaction (12 h) ($H_2Ti_2O_5{\cdot}H_2O$ on Ti felt), and (c) after the second-step reaction (TiO_2/Ti-F, first step: 12 h).

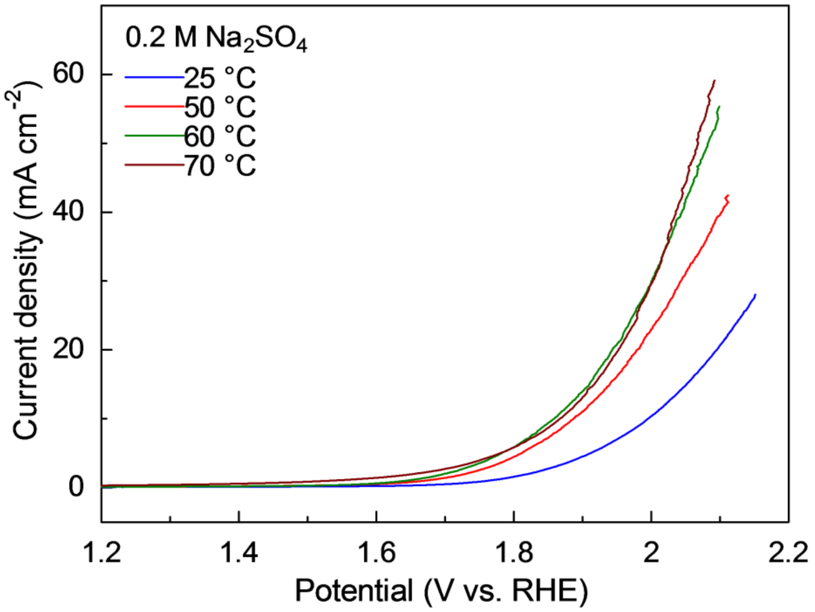

Figure S12. LSV curves of the IrO_2/C anode at (blue) 25, (red) 50, (green) 60, and (brown) 70 °C.

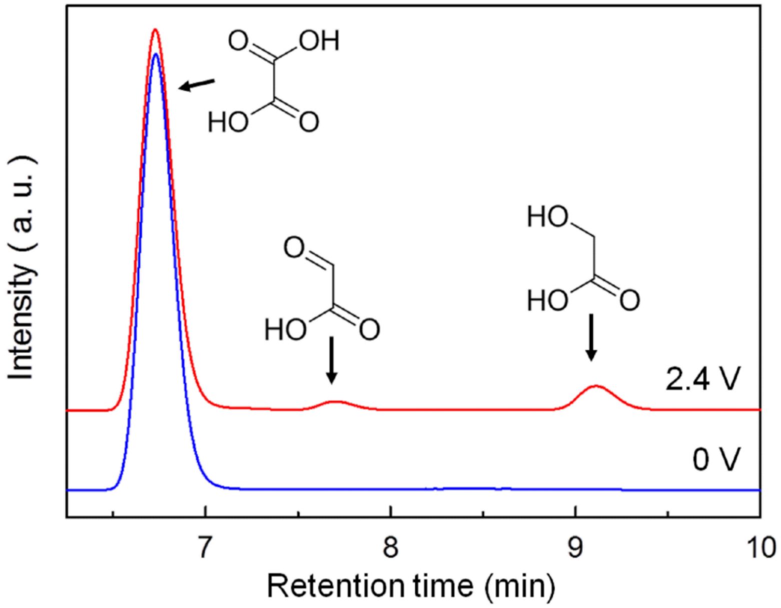

Figure S13. HPLC charts of downstream solution of the PEAEC (blue) before and (red) after applying voltage (2.4 V) at 25 °C.

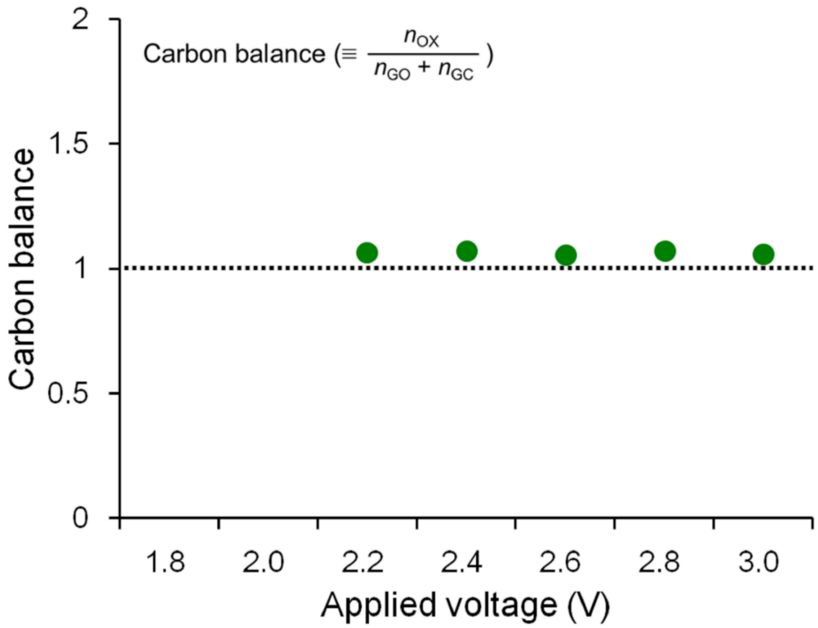

Figure S14. The carbon balance among OX, GO, and GC in the product solution at 25 °C.

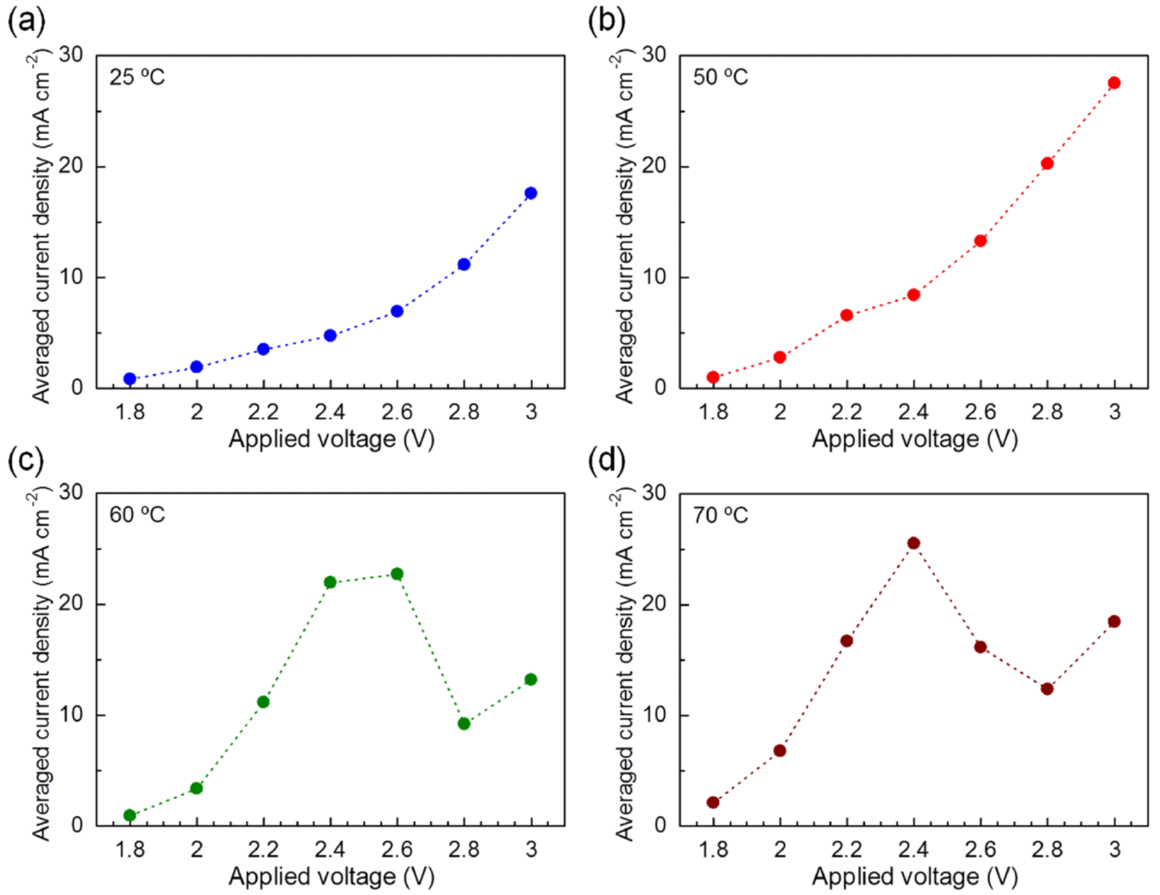

Figure S15. Averaged current density of the PEAEC during the operation at each applied voltage (reaction area: 4 cm^2, OX concentration: 0.03 M, flow rate: 0.5 ml min^{-1}).

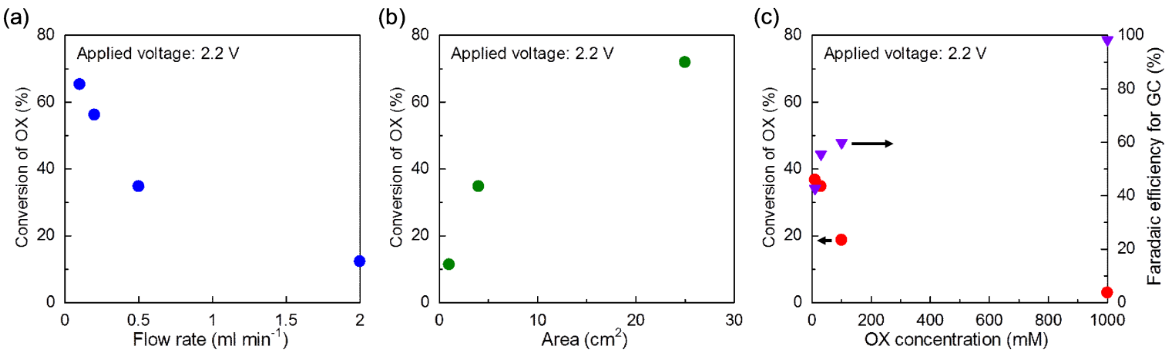

Figure S16. Dependences of OX conversion or Faradaic efficiency on (a) flow rate of reaction solution (reaction area: 4 cm^2, OX concentration: 0.03 M), (b) reaction area (flow rate: 0.5 ml min^{-1}, OX concentration: 0.03 M), and (c) OX concentration (reaction area: 4 cm^2, flow rate: 0.5 ml min^{-1}) under the operation of the PEAEC at 60 °C.

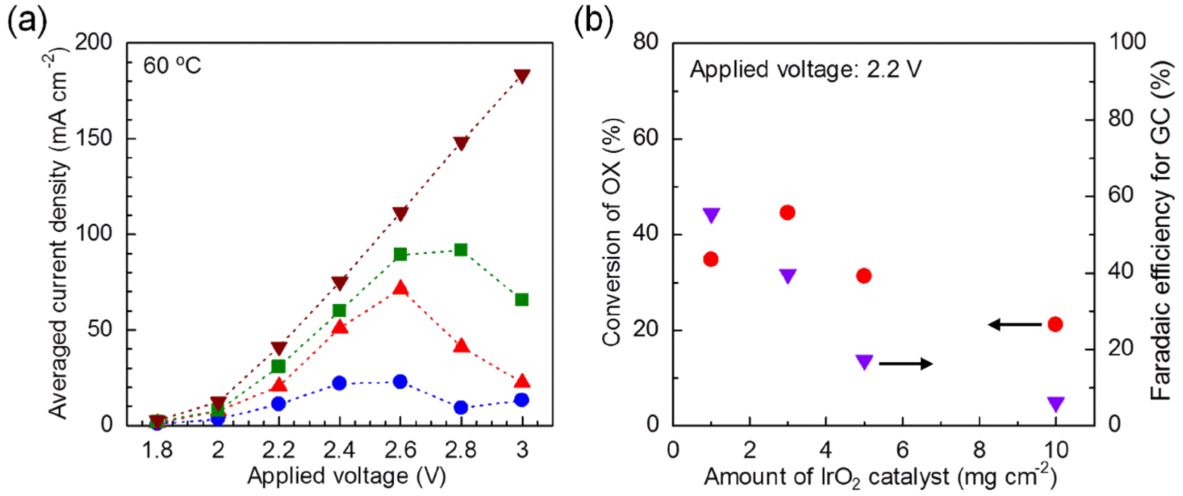

Figure S17. (a) Averaged current density of the PEAEC at 60 °C with (blue) 1, (red) 3, (green) 5, and (brown) 10 mg cm^{-2} of IrO_2 catalysts on anode (reaction area: 4 cm^2, OX concentration: 0.03 M, flow rate: 0.5 ml min^{-1}). (b) Dependence of conversion of OX and FE for GC (at 2.2 V applied voltage) on the amount of IrO_2 catalysts on anode.

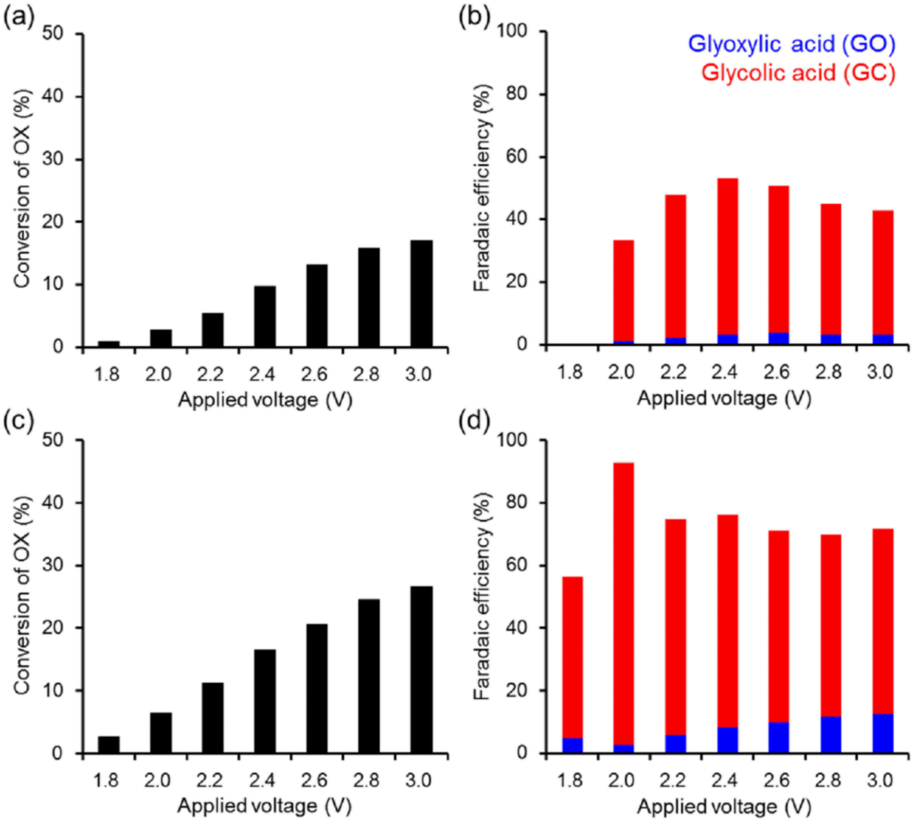

Figure S18. Performances of the PEAEC at 60 °C with TiO_2/Ti-M or TiO_2/Ti-F cathode (reaction area: 4 cm^2, flow rate: 0.5 ml min^{-1}, OX concentration: 1 M). (a) Conversion of OX and (b) Faradaic efficiency in the case of TiO_2/Ti-M. (c) Conversion of OX and (d) Faradaic efficiency in the case of TiO_2/Ti-F.
